# Examining the Impact of Extended Nursing Staff Work Hours on Nursing Home Costs

**DOI:** 10.1093/geroni/igaf122.1498

**Published:** 2025-12-31

**Authors:** Akbar Ghiasi, Rohit Pradhan, Justin Lord, Robert Weech-Maldonado

**Affiliations:** University of the Incarnate Word, San Antonio, Texas, United States; Texas State University, San Marcos, Texas, United States; Louisiana State University Shreveport, Shreveport, Louisiana, United States; The University of Alabama at Birmingham, Birmingham, Alabama, United States

## Abstract

Nursing home (NH) care is labor intensive with total labor costs constituting nearly 70% of an average facility’s operating costs. This study aimed to assess the relationship between nursing staff [registered nurses (RNs), licensed practical nurses (LPNs), certified nursing assistants (CNAs)] extended work hours and NH financial performance, specifically their operating costs. The study utilized four datasets: Care Compare: Five-Star Quality Reporting System, Medicare Cost Reports, LTFocus.org, and the Area Health Resource Files (2020-2022). A multivariable linear regression model with two-way fixed effects (year and state) was employed to analyze the data (N = 38,966). Separate regression models were estimated for RNs, LPNs, and CNAs. The dependent variable was operating costs, which include all costs incurred in direct resident care. The independent variable was extended work hours, measured as the percentage of nursing staff exceeding 50 work hours per week, averaged across the year to calculate the annual facility-level rate. After controlling for appropriate organizational and environmental level factors, the analysis did not find significant association between nursing staff extended work hours and NH operating costs. The findings suggest that extended work hours may be a convenient strategy for NH administrators to meet resident care demands, as it does not appear to significantly impact operating costs in our study. However, relying on extended hours could have unintended consequences, such as increased nursing staff burnout, reduced job satisfaction, and potential declines in care quality. While this approach may seem financially neutral, its long-term effects on resident outcomes and workforce stability warrant closer attention.

